# Ultraviolet Lithography-Based Ceramic Manufacturing (UV-LCM) of the Aluminum Nitride (AlN)-Based Photocurable Dispersions

**DOI:** 10.3390/ma13194219

**Published:** 2020-09-23

**Authors:** Paulina Ożóg, Paweł Rutkowski, Dariusz Kata, Thomas Graule

**Affiliations:** 1 Laboratory for High Performance Ceramics, Empa Swiss Federal Laboratories for Materials Science and Technology, Überlandstrasse 129, 8600 Dübendorf, Switzerland; thomas.graule@empa.ch; 2 Faculty of Materials Science and Ceramics, AGH University of Science and Technology, al. Adama Mickiewicza 30, 30-059 Cracow, Poland; Pawel.Rutkowski@agh.edu.pl (P.R.); kata@agh.edu.pl (D.K.)

**Keywords:** aluminum nitride, additive manufacturing, ceramics, LCM, UV-curing

## Abstract

In this work, three-dimensional (3D) shaping of aluminum nitride (AlN) UV-curable dispersions using CeraFab 7500 device equipped with the light engine emitting 365 nm wavelength (a UV-LCM device) is presented. The purpose of this study was the shaping of AlN pieces with microchannels for the future potential use as microchannel heat exchangers. The dispersions were characterized by the means of the particle size distribution, rheological measurements, and the cure depth evaluation. In shaping via UV-LCM, we applied dispersions containing 40 vol % solid load and different types of photoinitiators and their concentrations, as well as different settings of the printing parameters. Cuboidal plates with channels and cylindrical 3D structures were fabricated, debound, and sintered. For comparing ceramics properties, reference samples were prepared via uniaxial and cold isostatic pressing, using the same powder mixture as in the dispersions, and later sintered. The thermal conductivity of the sintered specimens was calculated, based on density and thermal diffusivity measurements.

## 1. Introduction

Aluminum nitride is an interesting and valuable material due to the attractive combination of properties, i.e., high thermal conductivity and wide bandgap (6.2 eV). It is mainly applied in the area of substrates for high-density integrate circuit packages [1,2,3,4]. Although AlN possesses several positive features, it also has some negative ones, which hinder the processing possibilities. It is a hygroscopic material requiring special caution regarding storage as the powder easily reacts with the moisture in the air [5,6]. Aluminum nitride exhibits strong covalent bonding and a low diffusive nature, so its densification is challenging and requires sintering temperatures exceeding 1900 °C to sinter it without the addition of any additives [7]. Therefore, sintering additives such as CaO, CaCO_3_, CaC_2_, MgO, Y_2_O_3_ [8,9,10,11,12] have to be applied. The presence of oxygen in AlN, in the form of Al_2_O_3_ on the powder’s surface and in a dissolved form in the AlN lattice [13,14,15] decreases the thermal conductivity of AlN. Therefore, aluminum nitride is an attractive material, however, its fabrication proves to be challenging.

Conventional ceramic shaping techniques, such as pressing, extrusion, and injection molding have some geometrical limitations. Additionally, these methods are not cost effective and require effort to produce small series of products due to the necessity of expensive mold production. The solution for overcoming such constraints is the use of additive manufacturing (AM) technologies [16]. The most prominent advantage of applying AM technologies in the field of ceramic materials is the possibility of shaping complex objects in a single process. Ceramics can be fabricated via various AM technologies, e.g., photopolymerization-based ones. Currently, the most popular materials used in those additive manufacturing technologies are mainly oxides, i.e., alumina [17,18,19,20,21,22,23], zirconia [24,25,26], and tricalcium phosphate [16]. Therefore, introducing AlN-based UV-curable dispersions to the field of additive manufacturing prove to be highly beneficial. This approach would provide the possibility of fabricating AlN pieces of high-resolution micro features, e.g., microchannel heat exchangers. Microstructural heat exchangers have a few advantages over conventional ones. They have a much higher ratio between the inner surface and volume, resulting in a significantly higher specific heat transfer surface. The values of heat and mass transfer coefficients are much higher for micro-channel devices exceeding those of conventional ones by one to two orders of magnitude [27]. Fabrication of micro-heat exchangers by additive manufacturing would replace the necessity of applying micro-machining, which is required for processing using conventional shaping methods, such as diffusion bonding or diamond tool milling/grinding [28].

In this work, the experience gained in UV-curable tape casting of aluminum nitride dispersions [29] is used as a base for the chosen AM method, which is lithography-based ceramic manufacturing (LCM). This paper is focused on the shaping process of AlN slurries and up to authors’ knowledge, this is the first presented approach to shaping such suspensions via LCM applying UV light by the light engine emitting 365 nm wavelength. Some guidelines for the preparation of AlN photoreactive suspensions are presented, as well as conducting UV-LCM, characterizing the liquid formulations, and describing the green body fabrication and sintering processes.

## 2. Materials and Methods

### 2.1. Raw Materials

As the main slurry constituent, aluminum nitride (AlN) powder grade B (BET = 1.85 m^2^∙g^−1^) from H.C. Starck (Goslar, Germany) was used. Yttrium oxide (Y_2_O_3_) powder (99.99 %, BET = 8.74 m^2^∙g^−1^) from Auer Remy (Hamburg, Germany) and aluminum oxide (Al_2_O_3_) TM-DAR (BET = 11.50 m^2^∙g^−1^) from Taimei Chemicals CO. Ltd. (Tokyo, Japan) were used as sintering additives to cause liquid phase formation during the sintering process. As a dispersing agent, glyceryl trioleate (GTO) from Sigma Aldrich (Buchs, Switzerland) in the concentration of 0.5 wt % (in respect to the powder weight) was used. It had a molecular weight of 885.43 g∙mol^−1^ and the density equal to 0.91 g∙cm^−3^. Additionally, another dispersant BYK-W 9010 (BYK) from BYK Additives & Instruments (Wesel, Germany) was used [29]. BYK-W 9010 is a phosphoric acid ester of a monofunctional carboxylic acid. It consisted of 100% active substance, had a molecular weight Mw ≈ 1000 g∙mol^−1^ and a chain length of 5–10 nm. Miramer M200 (1,6-hexanediol diacrylate (HDDA)) from Miwon Specialty Chemical CO., LTD. (Suji-gu, Korea) was used as a photocurable reactive binder. It is a liquid difunctional monomer with a viscosity between 0.005 and 0.015 Pa∙s at 25 °C and a refractive index of 1.455. As the photoinitiators, Genocure*ITX and Genocure*TPO-L from Rahn AG (Zürich, Switzerland) were used. Genocure*ITX (ITX) is isopropylthioxanthone in the form of a pale yellow solid, had a purity of ≥98% and exhibited strong absorption at the wavelength of 380 nm. It was combined with N, N, N′, N′-tetramethylethylendiamine (TEMED) (purity ≥99.5%) from Sigma Aldrich (Munich, Germany), which served as a co-initiator. Genocure*TPO-L (TPO-L) is ethyl (2,4,6-trimethylbenzoyl) phenyl phosphinate in the form of a yellow liquid with purity ≥93% and maximum absorbance at 370 nm.

### 2.2. Powder Surface Treatment with Dispersant and Reactive Dispersion Preparation

The first study on the development of the AlN-based UV-curable dispersion, published elsewhere [29], contains the description of the two-step treatment for powder surface modification, the so-called pre-treatment. The procedure involved coating powder surface with a dispersant in MEK/EtOH mixture, followed by solvent evaporation and redispersing the powder in a polymerizable binder. Yet, at that time, only AlN powder was incorporated in the dispersion preparation process. In the present study, Al_2_O_3_ and Y_2_O_3_ powders—which are used as sintering additives—were also used. Their surfaces were modified in the same way as AlN. After 24 h of ball milling separately, the powders were ball milled together for 1 h to homogenize the mixture. The final mass ratio (wt %) of AlN, Y_2_O_3_, and Al_2_O_3_ powders was 90:6:4. Next, they were dried under vacuum to be further redispersed in the reactive binder, and a UV-curable dispersion was prepared, used to fabricate three-dimensional objects by UV-LCM.

### 2.3. Three-Dimensional Shaping via LCM

A customised LCM device, CeraFab 7500 from Lithoz GmbH (Vienna, Austria) was used to fabricate three-dimensional structures. It operates with a UV light source emitting a wavelength of 365 nm and has a lateral resolution of 20 µm. The possible layer thickness is in the range of 10–100 µm. A layer thicknesses of 10 and 25 µm, and the exposure intensity of 19 mW∙cm^−2^ were applied during the shaping process. Given exposure intensity was used in each set of the printing parameters. Applying different exposure times resulted in a variety of exposure energies and printing parameter sets. Table 1 collects printing parameter sets used in this work. For the first five layers, higher exposure energy was applied to improve the adhesion of the shaped object to the building platform.

**Figure 1:**
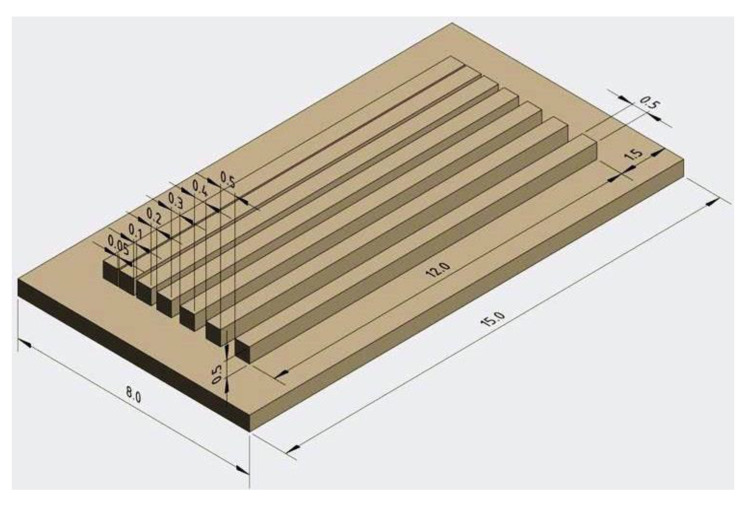
Design of the plate with channels (all dimensions in mm).

An excess of the unpolymerized slurry was removed using compressed air and a commercial cleaning solution LithaSol 20 from Lithoz GmbH (Vienna, Austria). To test the printing parameters settings, cuboidal specimens (5 mm × 5 mm × 1 mm) were fabricated. A plate with the bars separated by channels of the width 0.05 to 0.5 mm was shaped to test the possibility of microchannel fabrication using the UV-LCM device. This design is presented in Figure 1.

### 2.4. Reference Samples Preparation

Conventional pressing methods, i.e., uniaxial pressing and cold isostatic pressing were used to fabricate specimens from the powder mixture consisting of AlN, 6 wt % Y_2_O_3_ and 4 wt % Al_2_O_3_ (mass ratio 90:6:4). Uni-axial pressing was performed using a 10 mm-die, PW 20 press from Paul-Otto Weber Maschinen-Apparatebau GmbH (Remshalden, Germany) and applying the pressure of 136 MPa. The specimens were additionally densified via cold isostatic pressing (CIP) at the pressure of 2000 bars and dwell time of 60 s using CIP 400-76*200Y from EPSI (Temse, Belgium). Prior to CIP, pressed pellets were placed in medical protection Ultra-Cover 20 mm × 100 mm from Aichele Medico AG (Aesch, Switzerland) then degassed with a vacuum pump and tied tightly, to protect them during cold isostatic pressing (CIP).

### 2.5. Debinding and Sintering

The debinding process was performed in a furnace equipped with a gas flow system, under nitrogen atmosphere. After debinding, aluminum nitride specimens were embedded in a BN/AlN powder bed inside a graphite crucible. A graphite foil was placed on the top and the bottom of the powder bed to secure it during furnace evacuating. Sintering of AlN samples, prepared both by pressing and UV-LCM, was performed in a furnace hot press, applying no pressure, from Thermal Technology LLC High-Temperature Experts (Santa Rosa, CA, USA). Sintering was performed at a temperature of 1800 °C and a dwell time of 1h, under a nitrogen atmosphere, using 99.99% purity gas. The details of the debinding steps are given in Table 2.

### 2.6. Characterization Techniques

The evaluation on the effective dispersant concentration was presented in our previous study [29], using BYK-W 9010 and glycerol trioleate (GTO) as two dispersing agents. In the present study, GTO was used as the main dispersing agent and 0.5 wt % concentration was used to coat the powders and the resulting particle size distribution (PSD) was examined on LS 13 320 from Beckman Coulter (Krefeld, Germany). The PSD specimens were extracted directly from powder dispersions in MEK/EtOH, after mixing them for 24 h (so-called pre-treatment). All PSD measurements were performed in isopropyl alcohol and using Fraunhofer optical model.

Rheological measurements on ceramic dispersions were performed on a Modular Compact Rheometer MCR 302 from Anton Paar (Buchs, Switzerland) and using a coaxial cylinder measuring system CC27-SN36412. The effect of the solid content on AlN dispersions is given in [29]. The viscosity, as a function of the shear rate, was measured for dispersions containing 40 vol % of AlN powder and dispersions containing 40 vol % of solid fraction consisted of AlN, Y_2_O_3_ and Al_2_O_3_ (mass ratio 90:6:4 wt %), to evaluate the impact of the sintering additives on suspension viscosity.

The cure depth of AlN-based dispersions was evaluated by using CeraFab 7500 from Lithoz (Vienna, Austria) by photopolymerising 10 mm disc-specimens on the vat, applying different exposure energy values and measuring the discs’ thicknesses. The thickness of each disc was measured three times and an average was calculated. Dispersions containing 40 vol % solid loading and 0.5 wt % ITX combined with 0.25 wt % TEMED; and 1.0 wt % ITX with 0.5 wt % TEMED, and 0.5 wt %, 1.25 wt %, and 2.0 wt % TPO-L were examined.

Three-dimensionally shaped objects were analyzed under a light microscope SteREO Discovery V20 from Carl Zeiss (Oberkochen, Germany). Captured images were analysed using Imagic ims Client software from Imagic Bildverarbeitung AG (Glattbrugg, Switzerland).

Scanning electron microscope observations were carried out on NOVA NANO SEM 200 from FEI EUROPE using a backscatter electrons detector (BSED). Prior to SEM observations, the AlN sintered specimens were embedded in a resin, polished, and carbon sputtered.

Sintered AlN ceramic pieces were characterized by the means of apparent density using Archimedes principle, XRD crystal phase analysis on Panalytical MRX 4 from PANalytical (Herrenberg, Germany) with Excelerator Detector, and thermal diffusivity measurements. Thermal diffusivity of specimens prepared via pressing techniques and UV-LCM was measured on LFA 427 from Netzsch (Selb, Germany) equipped with the graphite sample holder. Measured values were used to calculate thermal conductivity according to Equation (1)

(1) $$\lambda \left(T\right)=a\left(T\right)\times \rho \left(T\right)\times c_{p}\left(T\right)$$

where *λ*—thermal conductivity [W∙m^−1^∙K^−1^], *ρ*—apparent density [g∙cm^−3^], *c_p_*—specific heat [J∙g^−1^∙K^−1^], and *a*—thermal diffusivity [mm^2^∙s^−1^]. The theoretical value of specific heat equal to 740 J∙kg^−1^∙K^−1^ (at the room temperature) [30] was used in the calculations of *λ*(*T*). 

## 3. Results and Discussion

### 3.1. Dispersion Preparation and Characterization

**Figure 2:**
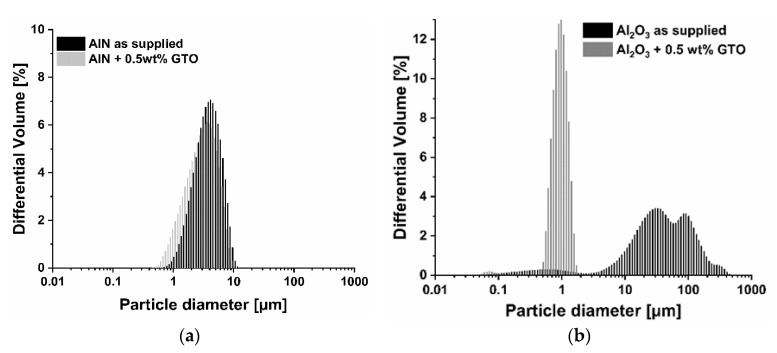

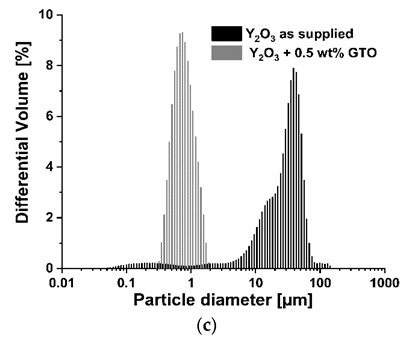
Particle size distribution of (**a**) AlN, (**b**) Al_2_O_3_, (**c**) Y_2_O_3_ treated with 0.5 wt % of GTO.

In the previously published study, 0.5 wt % of GTO was recognized as the lowest and efficient dispersing agent concentration for aluminum nitride-based slurries. Therefore, it was applied in the present study to break down the agglomerates of AlN powder and powders used as the sintering additives. Table 3 collects the values of *d*_10_, *d*_50_, *d*_90_ obtained in particle size distribution measurements for dispersions with (+) and without (−) the addition of a dispersant. It can be seen that *d*_10_, *d*_50_, and *d*_90_ were shifted towards lower values, which corresponded with the agglomerates breakage. Additionally, the PSDs of the powders are depicted in the graphs given in Figure 2. In the case of AlN, there was a small PSD change, whereas Al_2_O_3_ and Y_2_O_3_ powders exhibited a strong shift of the particle size towards a lower size range.

The values given in Table 3 and PSDs presented in Figure 2 indicate that the as supplied Al_2_O_3_ and Y_2_O_3_ powders were strongly agglomerated. The addition of GTO combined with the wet ball milling process was able to improve the dispersion condition by breaking the agglomerates and, in turn, reducing *d*_50_ from approx. tens of microns to below 1 µm and providing monomodal particle size distribution.

Surface modified powders were further used in the reactive binder, HDDA. In the previous study [29], the rheological study on the solid content was performed, however, without including sintering additives. In the present study, 40 vol % dispersion was enriched in 6 wt % Y_2_O_3_ and 4 wt % Al_2_O_3_ with respect to the mass of AlN. According to the literature, Y_2_O_3_ is the most effective sintering additive [5]. It reacts with Al_2_O_3_ located on the AlN grains surface to form a liquid phase, preferably the YAG phase, and removes the oxygen from the AlN lattice [9]. In our study, applying Y_2_O_3_ and Al_2_O_3_ may lead to an excessive formation of the liquid phase; however, it was done to promote material densification. 

**Figure 3:**
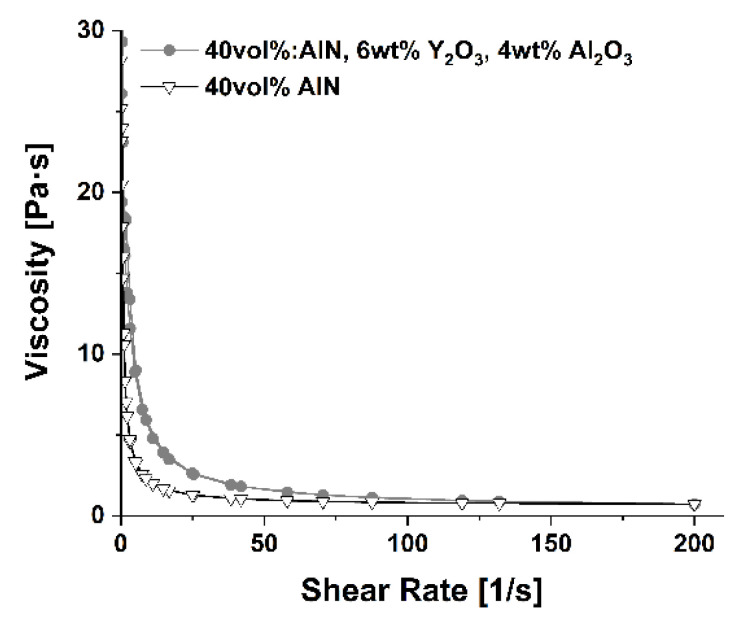
Viscosity comparison of 40 vol % dispersion with and without sintering additives.

The viscosity of the dispersion containing 0.5 wt % GTO and 40 vol % solid loading was examined as the function of shear rate and compared with the viscosity of the dispersion without sintering additives used in the previous study [29]. Both graphs are depicted in Figure 3. In both cases, slurries exhibited a shear thinning behavior. The addition of sintering additives slightly increased slurry viscosity, which is visible at shear rates up to 50 s^−1^. At shear rates exceeding 75 s^−1^, suspensions reach almost identical viscosity values. An increase in viscosity can be assigned to the fact that the sintering additives had smaller sized particles compared to those of AlN. Therefore, they filled the gaps between the coarser ones and created more contact surfaces which lead to a friction increase between all particles present in the dispersion.

**Figure 4:**
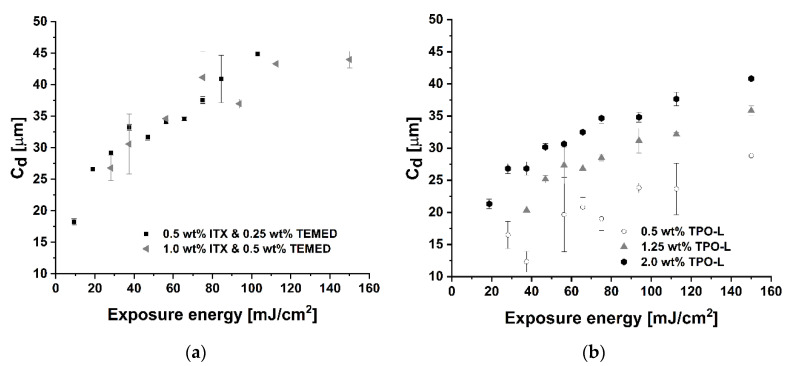
Cure depth in the function of exposure energy for slurries containing (**a**) ITX and TEMED, (**b**) TPO-L as a photoinitiator.

The cure depth of the ceramic dispersions plays a significant role in the shaping of three-dimensional objects via LCM, digital light processing, and stereolithography. In this study, the cure depth was examined for AlN dispersions prepared using ITX, together with TEMED and TPO-L at different concentrations, and the results are depicted in Figure 4. Solutions containing different amounts of ITX combined with TEMED (1:0.5 wt % and 0.5:0.25 wt %) exhibited similar cure depth values, independent of the applied concentrations. The depth of cure values increase parabolically and reach a maximum of 44 μm at the exposure energy of 150 mJ∙cm^−2^ for concentrations of 1.0 wt % ITX and 0.5 wt % TEMED. In the case of 0.5 wt % ITX combined with 0.25 wt % TEMED, the maximum cure depth of 45 μm was recorded at the exposure energy of 103 mJ∙cm^−2^. 

The dispersions containing different concentrations of TPO-L tended to polymerise thicker films by applying both higher exposure energy (i.e., ≥100 mJ∙cm^−2^) and higher photoinitiator concentration (i.e., 2 wt %). The maximum cure depth values of 29, 36, and 41 μm at the exposure energy of 150 mJ∙cm^−2^ were recorded for the TPO-L concentrations of 0.5 wt %, 1.25 wt %, and 2.0 wt % respectively.

Aluminum nitride dispersions exhibit low cure depth values compared to other ceramic materials, i.e., a cure depth in the range between 200 to 600 μm for 50 vol % silica dispersion [31], cure depth of 400 for 0.2 μm powder and 300 for 0.7 μm powder of 50 vol % alumina dispersion [32], and a cure depth range from 75 to 225 μm for 44 vol % zirconia slurry [24]. The poor cure depth of aluminum nitride dispersions could be caused by the high refractive index difference between AlN (*n* = 2.1) and HDDA (*n* = 1.455) being used as photocurable binder and by light absorption, which leads to the competition between light absorption of the AlN powder and photoinitiator. Additionally, aluminum nitride particles highly absorb and scatter light. That additionally contributes to the low cure depth values, which is similar as in the case of silicon nitride-based photocurable dispersions [32,33,34,35].

An examination of the polymer conversion degree may be an interesting completion of the cure depth study, to attain more comprehensive information about the photopolymerization of a ceramic dispersion. Such examination of photocurable AlN slurries can be found in our previous paper [29] and for other ceramic suspensions applicable in SLA—i.e., Chartier et al. [36] and Wu and Halloran [37].

### 3.2. Shaping of Three-Dimensional Structures

The solid loading of 40 vol % was used to prepare liquid formulations. Dispersions containing 1 wt % BYK-W 9010 as the dispersing agent was applied in UV-LCM, to be shaped using the CerFab 7500. However, the photopolymerization of a single layer was impossible. The dispersions tended to spread in reverse on the vat’s surface, and they were especially prone to do so when the dispersion layer thickness was set at the value below 100 μm. This was due to the too low suspension viscosity values (see Section 4.3. Effect of the Solid Load on the Viscosity in [29]). Such a problem was also mentioned in literature elsewhere [38]. However, too high dispersion viscosities might cause quality issues of 3D fabricated parts. This could be an issue particularly during the production of delicate geometries. During 3D printing, defects would be formed due to forces that are too high for the dispersion [39].

**Figure 5:**
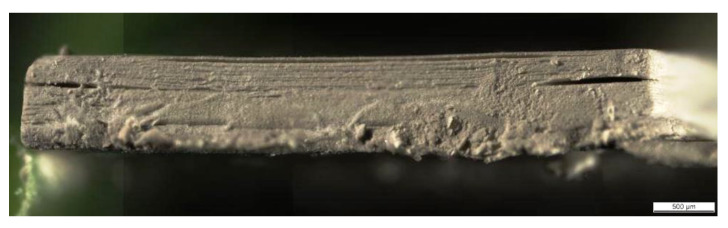
Side view of AlN green body fabricated from the dispersion containing 40 vol % of solid loading and 0.5 wt % ITX, 0.25 wt % TEMED.

Replacing 1.0 wt % BYK by 0.5 wt % GTO gave better results regarding the wetting of the vat’s surface. In the case of the photoinitiator, 1 wt % ITX and 0.5 wt % TEMED caused overpolymerization during a slurry solidification. Both concentrations were decreased to 0.5 wt % ITX and 0.25 wt % TEMED. That resulted in an improvement and enabled the fabrication of a cuboidal specimen, applying printing parameters PP-A. The side view of a cuboidal specimen is depicted in Figure 5. Some signs of overpolymerization were still visible and at the same time poor layers bonding, leading to delamination (arrows). The delamination could not be improved by changing the settings of the printing parameters. The shaping repeatability of this formulation was also limited.

The photoinitiator system consisting of ITX and TEMED was replaced by applying 0.5 wt % TPO-L. However, applying this TPO-L concentration and any exposure energy value listed in Table 1 resulted in the cure depth lower than the layer thickness of 25 μm used in the three-dimensional shaping. Despite that fact, applying printing parameters PP-B allowed to fabricate cuboidal specimens and a plate with channels.

**Figure 6:**
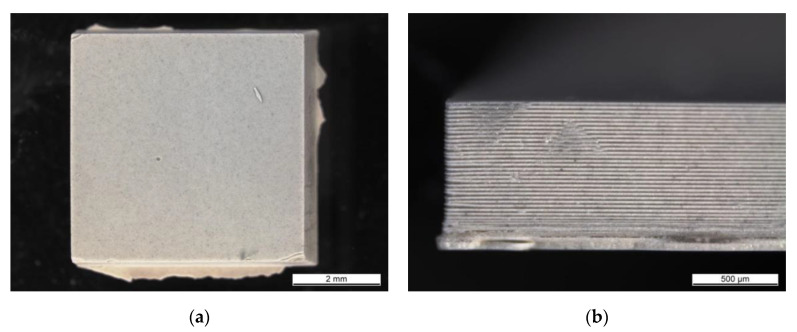
AlN green body 3D printed using printing parameters PP-A and 40 vol % dispersion and 0.5 wt % TPO-L. (**a**) top view, (**b**) side view.

Figure 6a shows the top view of a cuboidal specimen. A pinhole caused by an air bubble is present on the top of the part (an arrow). In the sample’s corners, some defects are present, caused by the peeling off of the edges (indicated by arrows). Changing the photoinitiator resulted in eliminating overpolymerization and higher quality specimen edges. Figure 6b depicts the side view of the part. Some layer delamination can be observed and the layer interface is visible. Both features are associated with insufficient cure depth. Proper bonding between layers is an important factor, as it further improves the structural properties of a final ceramic part. Therefore, it is necessary to ensure the sufficient depth of cure [40], i.e., in the recent work by Borlaf et al. [24] on zirconia, a cure depth of a value three times higher than the layer thickness was recommended to perform a successful shaping process by LCM. In our previous paper about tape casting of AlN-based photocurable slurries, limitations regarding cure depth of tapes were also observed. Attenuated total reflection-FTIR measurements and the following polymer conversion degree calculations showed differences in curing between two sides of the tapes; at the tapes’ top, the conversion degree was higher than on the bottom. The tape thickness also had an impact on the tapes curing behavior. The wrinkle formation appeared on the top of the tapes with thicknesses exceeding 100 μm. The cause was uneven polymerization through the tape’s thickness [29].

**Figure 7:**
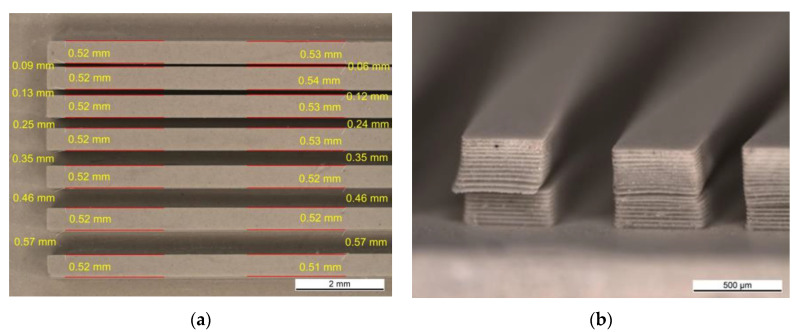
Plate with channels structure fabricated on the LCM device from a suspension containing 0.5 wt % TPO-L. (**a**) top view, (**b**) side view.

A structure called a plate with channels was obtained by using the same slurry as described above and the printing parameters PP-B. Despite low values of the cure depth, it was possible to fabricate that structure. Figure 7a shows the top view of one half of the plate with channels. The finest fabricated channel width was equal to 60 μm. In terms of the micro-heat exchanger fabrication, creating plenty of such channels would give an opportunity of increasing the surface area which would be in contact with the liquid, enhancing the heat transfer. Comparing the width of the physical object to those in the CAD model, an overgrowth of about 1.85% was observed. All the measured dimensions of the channel and bar widths are given in Figure 7a. The side view of the bars is given in Figure 7b. The photo revealed layer delamination as well as layer dislocation. Low cure depth values did not ensure sufficient bonding through the layers. Therefore, the adhesion forces between the cured layer and the vat’s surface could cause partial ripping off of a new layer from the preceding ones. Perhaps, low tilting speeds (5 steps∙s^−1^) had the benefit that the layers stuck together instead of completely separating from each other.

**Figure 8:**
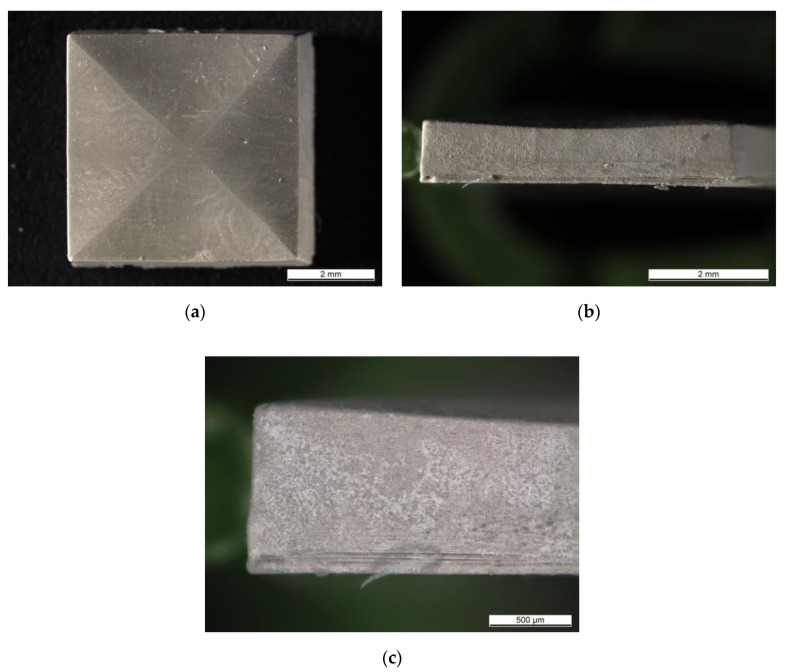
The structure fabricated on the LCM device from a suspension containing 0.5 wt % TPO-L and 10 µm printing layer thickness. (**a**) top view, (**b**) side view, (**c**) side view—higher magnification.

To improve the interface between the layers, the layer thickness printing parameter was decreased to 10 µm and AlN dispersion containing 0.5 wt % TPO-L was shaped applying the printing parameters PP-D (Table 1). Figure 8 shows photos of a fabricated cuboidal specimen; Figure 8a shows the top of the part and Figure 8b,c show the side of it. At the specimen’s top, the lines along its diagonals were observed as well as the brighter and shadowed areas. The location of those areas indicated that diagonal lines were the highest on the object’s top. Additionally, some dendrite-like patterns were observed next to diagonal lines. The side of the specimen (Figure 8b) was slightly bent towards its bottom surface (shown by the arrow). That was in a good correlation with the previous observations. In Figure 8c, some delamination was observed at the lower layers, close to the base of the specimen (which was in direct contact with the building platform). At the upper layers, no layer delamination was observed and the layer interface was not distinguishable. Decreasing the layer thickness printing parameter improved the interface between the layers.

**Figure 9:**
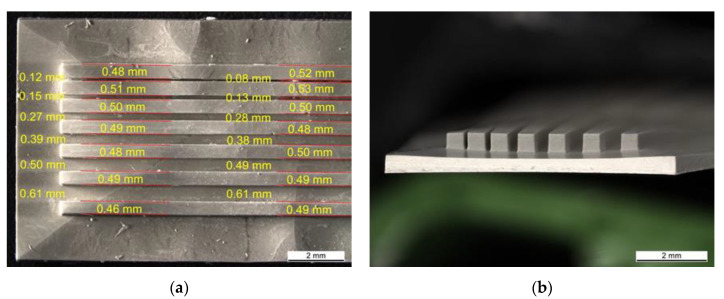
Plate with channels structure fabricated on the LCM device from a suspension containing 0.5 wt % TPO-L and 10 µm printing layer thickness. (**a**) top view, (**b**) side view.

The plate with channels structure was fabricated using 40 vol % solid load dispersion and 0.5 wt % TPO-L photoinitiator, and applying the printing parameters PP-D. It is shown in Figure 9. On the object’s top (Figure 9a) some brighter and shadowed areas, lines and a dendrite pattern scattered from those lines were observed. Such features could be caused by inhomogeneous powder distribution in the dispersion and/or by a wetting problem of the vat’s surface. The finest channel had a width of about 80 μm. The side view of the green body is given in Figure 9b, showing that no interface between the layers is visible.

In terms of *XY* plane resolution, the best results were obtained for the shaping dispersion containing 40 vol % solid loading, 0.5 wt % TPO-L photoinitiator, 25 µm layer thickness and the printing parameters PP-B (Table 1). In the case of *Z* plane resolution, the best features were recorded for decreasing the layer thickness printing parameter to 10 µm and applying the printing parameters set PP-D (Table 1).

**Figure 10:**
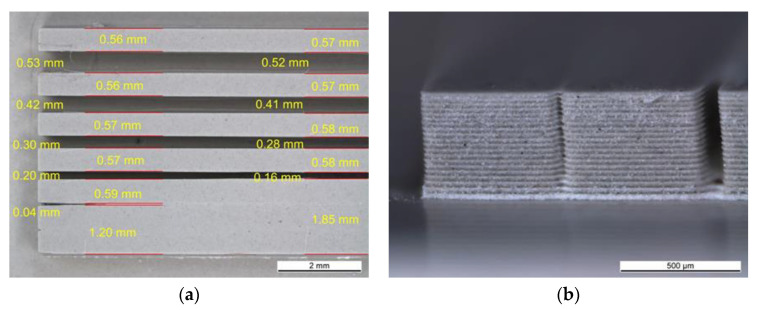
Plate with channel structure fabricated on the LCM device from a suspension containing 2.0 wt % TPO-L. (**a**) top view, (**b**) side view.

According to the cure depth evaluation (Figure 4), a higher photoinitiator concentration results in the polymerization of the thicker layers. The concentration of TPO-L was increased to 2 wt % and the printing parameters PP-C (Table 1) were applied to fabricate the plate with the channels. Figure 10 shows the photos of the part which was fabricated, Figure 10a is the top view on the half of the specimen and it shows the measured dimensions of the channels and the bars, and Figure 10b shows the view of the side of some bars. The layer interface could be distinguished and the finest channels (regarding the CAD design), of 50 and 100 μm width, were closed. By looking at the top of the object, one could expect that those fine channels were filled with the polymerised film. However, looking on the side of the specimen and measurements of the bars and channels widths suggested an overgrowth of the bars on the plate. Some gaps of about 59 μm were located on the end of the 100 μm channel. The 200 μm channel did not have a uniform width throughout its entire length. It was narrower in the middle than on the sides. Other channels exhibited similar features. The two phenomena could contribute to the dimension’s disproportions in *XY* plane in regard to the CAD design, i.e., overgrowth of the bars, and displacements of the layers along their length. With respect to the interface between the layers, it could be influenced by the low cure depth and the polymerization shrinkage of HDDA.

### 3.3. Debinding and Sintering of AlN Pieces

The LCM-shaped cylinder AlN specimens were prepared for the debinding and the sintering. For comparison, a pressed sample, consisting of the same powder mixture as the LCM shaped one (AlN and 6 wt % of Y_2_O_3_ and 4 wt % of Al_2_O_3_ as sintering additives) was prepared and directly sintered. Both debinding and sintering were performed under a nitrogen atmosphere.

**Figure 11:**
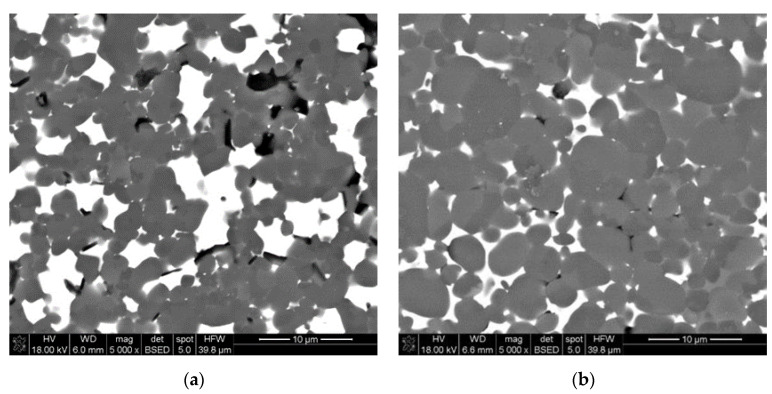
SEM images of sintered AlN specimens fabricated via (**a**) LCM, (**b**) uniaxial and cold isostatic pressing.

Three-dimensionally fabricated specimens had some micro-cracks after the debinding cycle. After sintering at 1800 °C for 1 h, the samples were polished, sputtered with carbon and imagined via SEM. In Figure 11a, the SEM image of the LCM-shaped sintered specimen is presented, and Figure 11b shows the image of the pressed sintered piece. In both cases, white areas corresponded to the liquid phase, dark grey areas were aluminum nitride grains and almost black areas could be pores remaining from the thermal treatment and/or defects caused by polishing, which were filled with another material, i.e., a sputtering layer material. In the case of both specimens, the liquid phase is not distributed uniformly. In Figure 11b, AlN grains with rounded grain boundaries, indicating their dissolution into the liquid phase, were not observed in the case of LCM-fabricated specimen.

The X-ray diffraction measurements (patterns not included) revealed the presence of aluminum nitride, yttrium-aluminum oxide (Y_3_Al_5_O_12_), boron nitride (remaining from the powder bed used during sintering) and aluminum-oxide-nitride in both kinds of specimens. Additionally, some yttrium oxide was detected in the sintered material prepared using own powder mixture shaped by LCM.

Properties, such as theoretical, apparent, and relative densities, as well as thermal conductivity of the sintered specimens fabricated via LCM and the pressing techniques, are collected in Table 4. The thermal diffusivity of both kinds of specimens was measured at 25 °C via the Laser Flash method. The thermal diffusivity values 2.06 and 31.39 mm^2^∙s^−1^ were recorded for LCM-shaped and pressed specimen, respectively. A theoretical value of specific heat equal to 740 J∙kg^−1^∙K^−1^ (at 25 °C) [30] was applied in calculations of thermal conductivity (λ_25 °C_).

The specimen shaped on the LCM-device exhibited lower apparent and relative densities than the pressed one. Furthermore, it had significantly lower thermal conductivity than the other specimen, originating from the high remaining porosity. In both cases, thermal conductivity is lower than the theoretical values given in literature, which is equal to a maximum value of 320 W∙m^−1^∙K^−1^ [1]. The amount of the used sintering additives (total 10 wt %), could drastically reduce the thermal conductivity of the rather dense pressed samples compared to the theoretical value. 

## 4. Summary and Outlook

In this study, it was shown that the shaping of AlN slurries via UV-LCM technology is challenging, especially at the curing stage. The dispersion wetting behavior on the vat’s surface is crucial for the shaping process. The cure depth should be higher than the printing layer thickness to ensure good bonding of the layers and avoid faults in the fabricated piece. The use of TPO-L as a photoinitiator resulted in parts with the best quality and better reproducibility of the LCM shaping process. Parts with the highest geometrical accuracy were fabricated using TPO-L concentration of 2.0 wt % and the channels with the width of 100 µm were fabricated. Applying a lower 0.5 wt % TPO-L concentration caused the occurrence of layer delamination. Decreasing the printing layer thickness from 25 to 10 µm improved layers adhesion and minimised the visibility of layer interfaces. After debinding and sintering the density of the LCM shaped parts were 2.84 g∙cm^−3^ (*ρ*_rel_ ≈ 84%) and the thermal conductivity was 4 W∙m^−1^∙K^−1^. Additional work is necessary to improve the formulations (solid loading increase, optimizing sintering additive amounts), the processing (increasing cure depth values and optimizing printing parameters to ensure good adhesion between layers) and optimize the debinding and sintering processes (obtaining dense, free of crack samples exhibiting high thermal conductivity values). Those improvements are necessary so that AlN slurries shaped by UV-LCM can be used to fabricate micro-heat exchangers.

## Figures and Tables

**Table 1:** Printing parameters (PP) used for shaping AlN three-dimensional structures

Printing Parameters	PP-A	PP-B	PP-C	PP-D
Layer Thickness (μm)	25	25	25	10
Exposure Energy (mJ∙cm^−2^)	66	38	47	47

**Table 2:** Debinding of aluminum nitride specimens shaped via LCM and conventional pressing

Heating Rate (min^−1^)	Temperature (°C)	Dwell Time (min)
-	25	-
0.1	120	360
0.5	360	960
0.5	460	360
Free cooling	25	0

**Table 3:** Particle size distribution of powders with (+) and without (−) dispersing agent applied

Material	0.5 wt % GTO	*d*_10_ (µm)	*d*_50_ (µm)	*d*_90_ (µm)
AlN	−	1.90	4.10	7.40
AlN	+	1.20	2.80	5.50
Al_2_O_3_	−	6.70	35.50	131.00
Al_2_O_3_	+	0.60	0.90	1.20
Y_2_O_3_	−	7.40	30.00	51.00
Y_2_O_3_	+	0.44	0.70	1.50

**Table 4:** Density values and thermal conductivity of the sintered ceramics.

Shaping Method	*ρ*_th_ (g∙cm^−3^)	*ρ*_app_ (g∙cm^−3^)	*ρ*_rel_ (%)	λ_25 °C_ (W∙m^−1^∙K^−1^)
UV-LCM	3.39	2.84	83.71	4.34
Pressing	3.39	3.27	96.38	75.96
